# Determinants of childhood stunting in the Democratic Republic of Congo: further analysis of Demographic and Health Survey 2013–14

**DOI:** 10.1186/s12889-017-4621-0

**Published:** 2017-08-01

**Authors:** Hallgeir Kismul, Pawan Acharya, Mala Ali Mapatano, Anne Hatløy

**Affiliations:** 1 Centre for International Health, University of Bergen, 5009 Bergen, Norway; 2 Nepal Development Society, Bharatpur, Chitwan Nepal; 3 Department of Nutrition, School of Public Health, University of Kinshasa, Kinshasa 1, Democratic Republic of Congo; 4 Fafo, Institute for Labour and Social Research, Box 2947 Toyen, 0608 Oslo, Norway

**Keywords:** Child health, Stunting, Chronic malnutrition, Growth disorders, Health status disparities, Social determinants, The Democratic Republic of Congo, DHS

## Abstract

**Background:**

Prevalence of child stunting in the Democratic Republic of Congo (DRC) is among the highest in the world. There is a need to systematically investigate how stunting operates at different levels of determination and identify major factors contributing to the development of stunting. The aim of this study was to look for key determinants of stunting in the DRC.

**Methods:**

This study used data from the DRC Demographic Health Survey 2013–14 which included anthropometric measurement for 9030 under 5 year children. Height-for-Age Z score was calculated and classified according to the WHO guideline. The association between stunting and bio-demographic characteristics was assessed using logistic regression.

**Results:**

Prevalence of stunting was much higher in boys than girls. There was a significant rural urban gap in the prevalence of stunting with rural areas having a larger proportion of children living with stunting than urban.

Male children, older than 6 months, preceding birth interval less than 24 months, being from lower wealth quintiles had the highest odds of stunting. Several provinces had in particular high odds of stunting. Early initiation of breastfeeding, mother’s age more than 20 years at the time of delivery had lower odds of stunting. The taller the mother the less likely the child was to be stunted. Similarly, mother’s BMI, access to safe water, access to hygienic toilet, mother’s education were found negatively correlated with child stunting in the bivariate logistic regression, but they lost statistical significance in multivariate analysis together with numbers of children in the family and place of residence.

**Conclusions:**

Child stunting is widespread in the DRC and increasing prevalence is worrisome. This study has identified modifiable factors determining high prevalence of stunting in the DRC. Policy implementation should in particular target provinces with high prevalence of stunting and address modifiable determinants such as reducing socioeconomic disparity. Nutrition promotion intervention, including early initiation of breastfeeding should be an immediate priority.

**Electronic supplementary material:**

The online version of this article (doi:10.1186/s12889-017-4621-0) contains supplementary material, which is available to authorized users.

## Background

The Democratic Republic of Congo (DRC) has among the highest percentages of chronic child malnutrition, or stunting, in the world with lasting consequences for the children who suffer from this nutritional disorder [1]. Stunting during early childhood has several negative implications and has been associated with adverse effects on cognitive development, school achievement and economic productivity in adulthood and maternal reproductive outcomes [2, 3].

Globally, there has been a significant decline in the prevalence of stunting below the age of 5 years. In 1990 as much as 40% of children were stunted but in 2015 the percentage had declined to 24% [1, 4]. While, the prevalence of stunting at the global level declined significantly, the decrease has been more modest in sub-Saharan Africa. Some sub-Saharan countries have experienced a reduction in stunting, other countries have not managed to combat stunting and in these countries chronic malnutrition is widespread with prevalence over 40%. The DRC is among these countries and has among the highest prevalence of stunting in the region [5].

Studies across geographical settings show that weight and length at birth and during the first 5 years of life are similar across different conditions when mother’s nutritional and health needs are fulfilled and when constraints on growth are limited [6]. In the same way, under such conditions the growth of children from different continents demonstrates very similar growth patterns [7, 8]. A number of factors may cause linear growth failure, including infections and suboptimal feeding practices. In addition, various environmental conditions can influence linear growth during the first years of life, including maternal nutritional status, access to safe drinking water, hygiene and sanitation. There is growing understanding of the relation between stunting and socio-economic factors. The literature has demonstrated social inequalities in nutrition, showing that children living in poor households are more likely to be stunted than children from richer households [9–11]. It has been suggested that economic growth in low-income countries is associated with a parallel improvement in children’s nutritional status [12, 13]. In order for economic growth to reduce stunting it needs to improve access to sanitation, better education for women, increase access to quality food and reduce social inequalities [14, 15].

There are several studies that have examined factors associated with child stunting including biological, demographic and social factors. However, there is a need for studies that further investigate how stunting operates at different levels of determination in order to identify key factors contributing to the development of stunting. In our study, we apply a conceptual framework for determinants of malnutrition with the intention of comparing the importance of determinants at different levels.

There are to our knowledge only two studies that have analysed national data on factors determining children’s nutritional status in the DRC. A study used data from the 2001 DRC Multiple Indicators Cluster Survey (MICS) to examine the association between maternal education and child nutritional status [16]. A second study employed data from the 2007 DRC Demographic Health Survey (DHS) [17]. The study primarily addressed the issue of the impact of geographic location on children’s nutritional status. There is therefore no study that uses the more recent data from the DRC-DHS 2013–14 to examine the wide range of factors that contribute to the high prevalence of stunting in the DRC.

The aim of the study is to look for key factors associated with childhood stunting in the DRC. The study uses data from the DRC-DHS 2013–14 collected from the nationally representative cross-sectional survey conducted in 2013/14. By identifying key factors determining stunting the results from our study can be transferred to other settings than the DRC with the findings, especially, being relevant for nutritional initiatives in areas with severe problems of stunting.

## Methods

### Survey

**Fig. 1:**
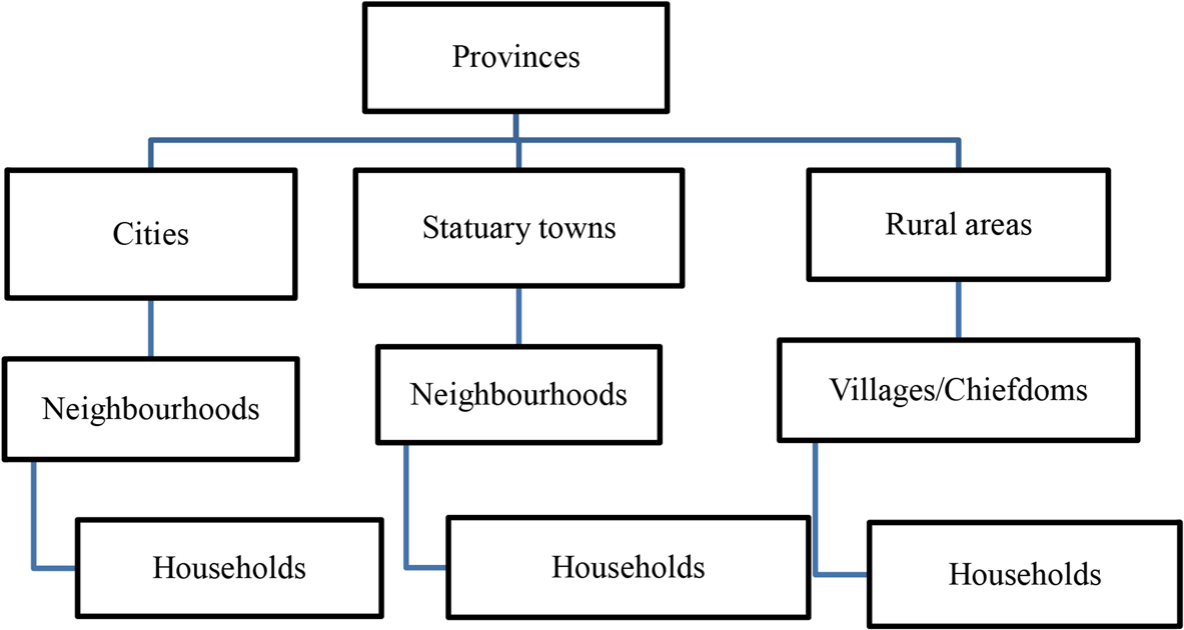
Multistage sampling procedure, DRC DHS 2013-14

We analysed data for children below the age of 5 years from the DRC-DHS 2013–14 conducted in 2013/14 [18]. The survey was conducted as a multistage cluster sample survey. In accordance with the DRC’s administrative division into provinces the survey divided the country into 26 sampling domains. These domains were further stratified into urban and rural areas. From the urban areas neighbourhoods were sampled from cities and towns whereas for rural areas villages and chiefdoms were sampled. Subsequently a fixed number of households were chosen from each of the selected clusters (Fig. 1). Details of the DHS methodology is elaborated in the measureDHS website [19] and the country specific methodology for the DRC DHS 2013–14 is elaborated in the final report (in French) which can be freely downloaded from the measureDHS website [20].

The DHS collected anthropometric data for all women and children younger than 5 years in the selected household. In total, 9369 children were measured for their anthropometric indices. Afterwards, based on WHO growth standards, 331 children were excluded because of the extreme HAZ value i.e. HAZ above 6SD or below -6SD and 44 children were excluded for biologically implausible heights, resulting in 8994 valid measurements. Children who did not sleep last night at their home were futher excluded (*n* = 110) from the analysis. Therefore, the total number of children included in this analysis was 8884 and after adjusting the cluster design and sample weight the corrected sample size became 9030. WHO child growth standard is a widely used criteria in data cleaning while analyzing anthropometric data [21]. A study by using data from 21 DHS countries found WHO standard 2006 as the most inclusive criteria resulting in the highest reported prevalence compared to other standards [22]. Since 2006, DHS is using the WHO 2006 criteria as it started using the WHO 2006 reference population to compute the z scores [23].

### Variables

#### Dependent variable

Data regarding the height/length and weight were obtained for children below 59 months of age. Height-for-age index was calculated according to the WHO-MGRS 2006 Child Growth Standards [21]. A z-score of height for age index (HAZ) < −2SD was defined as stunted. A HAZ between -2SD and -3SD was considered as moderate stunting, and HAZ < −3SD as severe stunting [24–26].

#### Independent variables

The information regarding the background bio-demographic and socioeconomic characteristics of children less than 5 years of age were obtained from interview with their mothers. Maternal nutritional status was assessed by BMI, and coded as follow: below 18.5 = malnourished; 18.5–24.9 = normal; and BMI ≥25.0 = overweight.

### Analytical framework

We modified the analytical framework from Hien and Hoa [27]. This framework was based on the UNICEF conceptual framework describing determinants as tiers of interrelations [28–30]. The UNICEF framework clarifies how the problem of child malnutrition is related to factors at higher levels and thereby views the problem of malnutrition as a larger development problem [31].

**Fig. 2:**
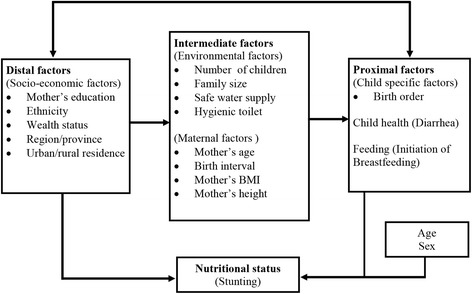
Conceptual framework for hierarchial regression modeling of determinants of child nutritional status

Based on the conceptual framework we grouped the independent variables into distal, intermediate and proximal factors (Fig. 2).

The distal factors included were mother’s education, ethnicity wealth quintile, region/province and urban/rural residence. Intermediate characteristics were: environmental factors such as number of children in the family, family size, availability of safe water supply and hygienic toilet, and maternal factors including mother’s age at delivery, preceding birth interval, mother’s BMI and mother’s height. We included birth order, had diarrhoea within the past 14 days and breastfeeding initiated within the first hour after birth as proximate factors. We kept age and sex separately because age and sex might work independently or through the distal, intermediate or proximal factors.

### Statistical analysis

The analysis was performed using STATA 14.0. At first, the data was set as survey data using “svyset” command by adjusting the cluster design and sample weights. Prevalence of childhood stunting was calculated using svy: command prefix [32]**.** Survey analysis technique is recommended for DHS data [33]. Differences in stunting prevalence across background characteristics were tested using chi-square test.

The association between bio-demographic and socioeconomic characteristics with child stunting were assessed by three different logistic regression models. Initially, child’s age, sex and distal factors were included in the model. In the second model, intermediate factors were added to see the effect of distal factors through the intermediate factors. Finally, in the third model child specific proximate factors were additionally adjusted to the regression analysis.

Additionally, a multinomial logistic model was used to estimate the relative risk ratio (RRR) for the severity of stunting according to the background characteristics. 

## Results

### Descriptive analysis

The prevalence of stunting was 42.7% [95% CI: 41.30, 44.15] comprising of 20.2% moderate and 22.5% severe stunting (Table 1).

**Table 1:** Sample distribution and prevalence of Stunting among under five year children; the Democratic Republic of Congo 2013–14

Characteristics	Sample		Moderate	Severe	Total Stunting(<−2SD)	
	%	n	Stunting %	Stunting %	%	95% CI
Age					*p* < 0.001	
< 6 months	10.8	976	8	6.7	14.7	[12.0,17.7]
6–11 months	9.8	888	9.9	13.2	23.1	[18.9,27.8]
12–23 months	18.9	1707	21.4	17.7	39.1	[35.9,42.3]
24–35 months	20.4	1840	23.4	26.8	50.2	[47.1,53.4]
36–47 months	19.7	1783	24.7	29.3	54.0	[50.7,57.3]
48–59 months	20.4	1838	23.2	28.9	52.1	[48.8,55.3]
Total	100	9030	20.2	22.5	42.7	[41.3,44.2]
Sex					*p* < 0.01	
Male	50.0	4517	20.4	24.9	45.2	[43.2,47.3]
Female	50.0	4513	20.1	20.1	40.2	[38.2,42.2]
Total	100	9030	20.2	22.5	42.7	[41.3,44.2]
Distal factors						
Mother's education					*p* < 0.001	
None	19.7	1641	20.1	30.6	50.8	[47.5,54.0]
Primary	43.7	3632	21.3	25.8	47.1	[44.8,49.4]
Secondary	35.4	2945	20.1	13.1	33.2	[30.9,35.6]
Higher	1.2	97	9.8	3.3	13.1	[7.3,22.5]
Total	100	8315	20.5	22.0	42.5	[41.0,44.0]
Wealth quintile					*p* < 0.001	
Richest	16.1	1456	14.0	9.0	22.9	[20.2,26.0]
Richer	18.5	1671	20.4	21.0	41.4	[37.9,45.0]
Middle	20.4	1843	22.3	23.5	45.8	[42.6,49.0]
Poorer	22.4	2018	22.2	26.1	48.3	[45.1,51.5]
Poorest	22.6	2042	20.8	28.8	49.7	[46.9,52.5]
Total	100	9030	20.2	22.5	42.7	[41.3,44.2]
Residence					*p* < 0.001	
Urban	30.2	2728	18.7	13.8	32.5	[30.3,34.7]
Rural	69.8	6302	20.9	26.2	47.2	[45.4,49.0]
Total	100	9030	20.2	22.5	42.7	[41.3,44.2]
Intermediate factors						
Mothers age at delivery					*p* = 0.084	
< 20 years	14.5	1307	24.0	22.5	46.5	[43.0,50.1]
20–34 years	63.2	5704	19.9	22.0	41.9	[40.1,43.7]
> =35 years	22.4	2020	18.9	23.8	42.6	[39.6,45.7]
Total	100	9030	20.2	22.5	42.7	[41.3,44.2]
Mother’s body mass index					*p* < 0.001	
Thin	11.8	944	22.7	22.8	45.4	[41.0,50.0]
Normal	72.6	5829	20.8	23.3	44.1	[42.4,45.9]
Overweight	15.6	1252	17.4	15.7	33.1	[29.5,36.8]
Total	100	8025	20.5	22.0	42.6	[41.1,44.1]
Birth interval					*p* < 0.001	
First Birth	18.1	1470	21.9	19.1	41.0	[37.7,44.4]
< 24 months	21.6	1755	18.9	29.4	48.3	[44.9,51.8]
24–47 months	47.35	3856	21.3	21.1	42.4	[40.3,44.6]
48 months or more	13.1	1063	17.8	17.0	34.7	[30.9,38.8]
Total	100	8143	20.4	22.0	42.4	[40.9,43.9]
Family size					*p* = 0.532	
Small(<4)	22.3	1711	21.6	20.3	41.8	[38.7,45.1]
Medium (4–6 members)	17.0	1303	19.3	21.7	41.0	[37.4,44.6]
Large (>8 members)	60.7	4660	19.2	24.0	43.2	[41.2,45.2]
Total	100	7674	19.7	22.8	42.5	[41.0,44.06]
Number of Children					*p* < 0.5	
1	20.0	1806	22.0	17.5	39.5	[36.5,42.7]
2	44.6	4028	20.7	23.2	43.9	[41.7,46.1]
3	26.5	2394	19.2	25.5	44.7	[41.9,47.5]
4 or more	0.9	79	17.7	21.6	39.3	[34.6,44.2]
Total	100	9030	20.2	22.5	42.7	[41.3,44.2]
Access to safe water		0			*p* < 0.001	
No	70.6	6372	21.5	25.0	46.5	[44.8,48.1]
Yes	29.4	2658	17.3	16.4	33.8	[31.4,36.6]
Total	100	9030	20.2	22.5	42.7	[41.3,44.2]
Access to hygienic toilet		0			*p* < 0.001	
No	96.5	8717	20.6	23.1	43.7	[42.2,45.2]
Hygienic	3.5	313	10.1	5.5	15.6	[11.0,21.6]
Total	100	9030	20.2	22.5	42.7	[41.3,44.2]
Proximal factors						
Birth order					*p* = 0.113	
1	18.0	1476	21.9	19.1	41.0	[37.7,44.4]
2	16.5	1352	20.5	19.2	39.7	[36.3,43.3]
> =3	65.5	5363	20.0	23.5	43.6	[41.7,45.5]
Total	100	8192	20.5	22.1	42.5	[41.0,44.0]
Had Diarrhoea					*p* = 0.441	
No	82.4	6691	20.6	22.2	42.8	[41.2,44.5]
Yes	17.6	1430	19.8	21.4	41.2	[37.7,44.9]
Total	100	8121	20.5	22.0	42.5	[41.0,44.0]
Breastfeeding within first hour					*p* = 0.120	
No	55.6	4459	22.1	21.5	43.6	[41.6,45.7]
Yes	44.4	3566	18.3	22.9	41.2	[39.0,43.4]
Total	100	8025	20.4	22.1	42.5	[41.0,44.1]

*p p*-value using chi-square test, *CI* confidence interval

**Fig. 3:**
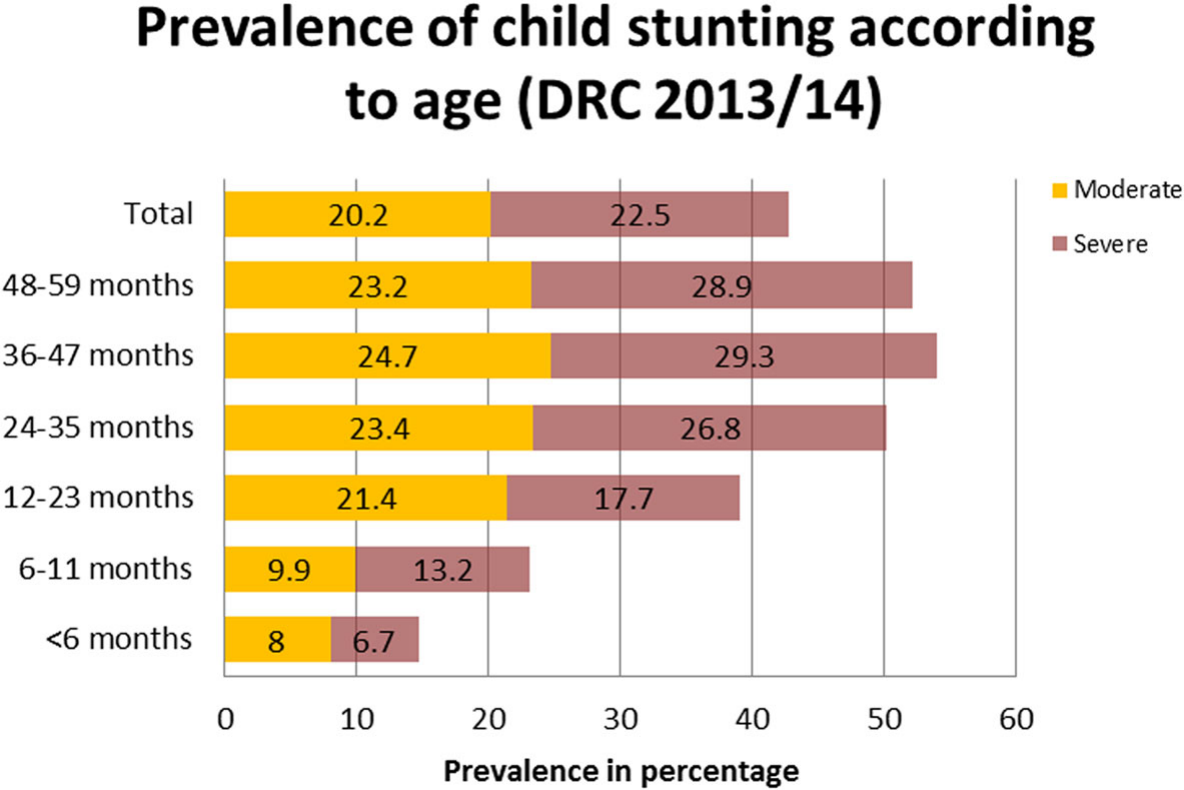
Prevalence of child stunting according to age (DRC 2013/14)

**Fig. 4:**
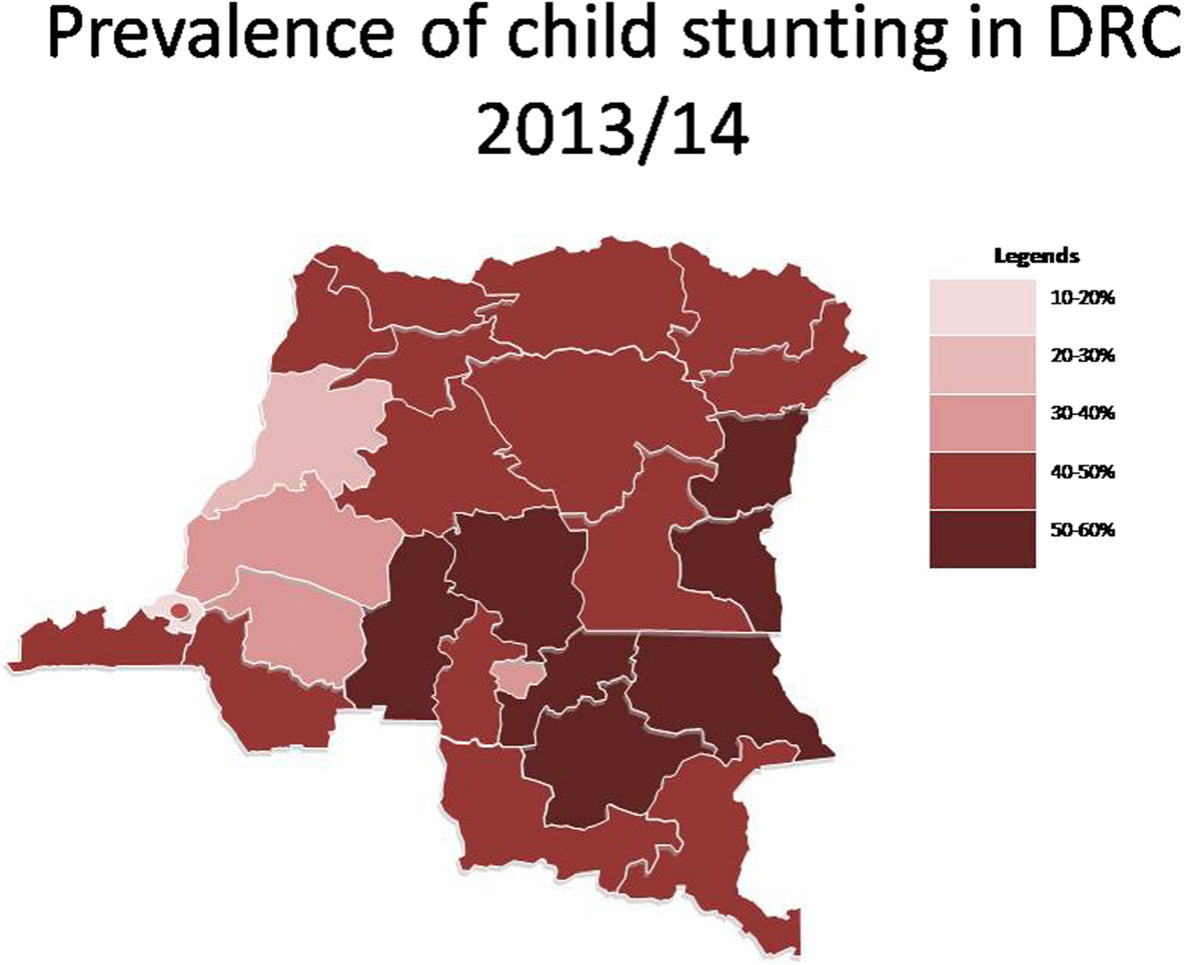
Prevalence of child stunting across the regions (DRC 2013/14)

Highest prevalence of stunting, 54.0%, was found in the age group 36–47 months (Fig. 3). Prevalence of stunting was significantly higher in boys than in girls with the figures being 45.2% for boys and 40.2% for girls. The prevalence was higher among the children of mothers with little or no education. The prevalence of stunting among the children whose mother’s didn’t have any school education was 50.8%, on the other hand, the prevalence was 13.1% among the children of mothers with higher education. The prevalence among the poorest wealth quintile was 49.7%, which declined to 22.9% among the richest wealth quintile. There was a significant rural urban gap in stunting with 47.2% of children living in rural areas being stunted versus 32.5% of children living in urban areas. According to the provinces the prevalence ranges from 17.3% in Kinshasa to 57.95% in Kasai (Fig. 4). Also, the prevalence of stunting according to the provinces is shown in the additional file (See Additional file 1: Table S1). Detailed distribution of stunting is shown in Table 1.

### Bivariate analysis

Bivariate logistic regression analysis showed that age and sex was associated with childhood stunting. Among the distal factors- mother’s education and wealth quintiles were found to be negatively correlated with child stunting. Rural residency and province were also associated with stunting. Intermediate factors including mother’s age at delivery, mother’s BMI, preceding birth interval, number of children in the household were also found to be associated with stunting together with access to safe water and access to hygienic toilet facility (See Additional file 2: Table S2).

### Hierarchical logistic regression analysis

#### Distal factors

Mother’s education was significantly associated in the first model where the aOR for highly educated mother’s children was 0.45 (95% CI 0.22, 0.93). In the following models the statistical significance was lost. In addition stunting was significantly associated with wealth quintile in all the three models. Children from the poorest wealth quintile had an aOR = 2.90 (95% CI 1.83, 4.54) for being stunted compared to children from the richest wealth quintile. Regarding the provinces, model-3 showed that children from Kasai, Tshuapa, Kasai-oriental, Sankuru, Haut-Katanga, and Sud-Kivu provinces had significant higher odds ratio of stunting in comparison to the children from Kinshasa province. With regards to residence, urban/rural residence, this variable was not statistically significantly associated with child stunting (Table 2).

**Table 2:** Hierarchical logistic regression analysis of associations between stunting and sociodemographic and clinical factors in under-five children, the Democratic Republic of Congo, 2013–14

Characteristics	Model 1				Model 2				Model 3			
	aOR	95% CI			aOR	95% CI			aOR	95% CI		
Age												
< 6 months	1.00				1.00				1.00			
6–11 months	1.89	1.12	3.19	*	1.84	1.07	3.19	*	1.84	1.09	3.13	*
12–23 months	4.22	3.07	5.80	***	4.35	3.14	6.03	***	4.41	3.19	6.09	***
24–35 months	6.59	4.84	8.97	***	6.34	4.60	8.74	***	6.53	4.73	9.00	***
36–47 months	7.93	5.77	10.89	***	8.78	6.41	12.03	***	9.60	6.97	13.21	***
48–59 months	7.67	5.61	10.50	***	7.63	5.59	10.42	***	8.13	5.93	11.15	***
Sex												
Male	1.00				1.00				1.00			
Female	0.80	0.70	0.92	**	0.81	0.70	0.94	**	0.81	0.70	0.94	**
*Distal factors*												
Mother's education												
None	1.00				1.00				1.00			
Primary	1.03	0.88	1.21		1.02	0.86	1.21		0.99	0.83	1.18	
Secondary	0.80	0.67	0.96	*	0.84	0.68	1.03		0.83	0.67	1.04	
Higher	0.45	0.22	0.93	*	0.64	0.31	1.32		0.63	0.31	1.28	
Wealth quintile												
Richest	1.00				1.00				1.00			
Richer	2.14	1.57	2.92	***	1.92	1.32	2.79	**	1.91	1.32	2.76	**
Middle	2.84	1.98	4.07	***	2.30	1.51	3.50	***	2.38	1.56	3.62	***
Poorer	3.28	2.23	4.83	***	2.43	1.54	3.82	***	2.48	1.57	3.92	***
Poorest	3.93	2.70	5.72	***	2.81	1.78	4.44	***	2.90	1.83	4.57	***
Ethnicity												
Bakongo	1.00				1.00				1.00			
Bas-kasai et kwilu-kwngo	0.93	0.52	1.67		0.82	0.45	1.49		0.92	0.51	1.66	
Cuvette central	0.77	0.41	1.45		0.60	0.31	1.14		0.65	0.34	1.24	
Ubangi et itimbiri	1.02	0.53	1.96		1.07	0.53	2.17		1.11	0.54	2.24	
Uele lac albert	1.08	0.52	2.24		0.99	0.45	2.18		1.05	0.48	2.30	
Basele-k, man. et kivu	0.96	0.45	2.05		0.91	0.41	2.01		0.93	0.41	2.11	
Kasai, katanga, Tanganyika	1.00	0.54	1.85		0.94	0.49	1.79		1.08	0.57	2.05	
others (lunda/pygmy/foreign)	1.28	0.62	2.68		1.23	0.60	2.50		1.61	0.83	3.13	
Residence												
Urban	1.00								1.00			
Rural	0.92	0.74	1.15		0.90	0.69	1.18		0.89	0.68	1.16	
Province (New)												
Kinshasa	1.00								1.00			
Kwango	1.81	0.93	3.50		1.62	0.78	3.36		1.42	0.69	2.91	
Kwilu	1.29	0.73	2.27		1.41	0.74	2.67		1.29	0.69	2.43	
Mai-ndombe	1.07	0.59	1.92		1.12	0.61	2.03		1.02	0.56	1.84	
Kongo central	2.09	0.97	4.48		1.64	0.72	3.72		1.77	0.84	3.77	
Equateur	0.58	0.27	1.24		0.60	0.26	1.40		0.55	0.24	1.28	
Mongala	0.96	0.52	1.79		0.84	0.41	1.73		0.79	0.39	1.61	
Nord-ubangi	1.22	0.60	2.51		1.33	0.61	2.91		1.23	0.56	2.69	
Sud-ubangi	1.22	0.62	2.42		1.00	0.48	2.08		0.95	0.46	1.96	
Tshuapa	1.71	0.86	3.41		2.54	1.24	5.18	*	2.33	1.15	4.72	*
Kasai	2.82	1.60	4.97	***	3.05	1.65	5.61	***	2.75	1.49	5.07	**
Kasai-central	1.89	1.02	3.49	*	1.74	0.88	3.46		1.48	0.74	2.96	
Kasai-oriental	1.80	1.03	3.15	*	2.05	1.14	3.70	*	1.86	1.04	3.33	*
Lomami	1.97	1.11	3.50	*	1.92	1.00	3.68	*P* = 0.05	1.64	0.86	3.11	
Sankuru	2.49	1.32	4.69	**	3.11	1.54	6.30	**	2.87	1.41	5.82	**
Haut-katanga	2.84	1.72	4.69	***	2.55	1.46	4.46	**	2.19	1.26	3.81	**
Haut-lomami	1.77	0.95	3.30		1.28	0.63	2.57		1.13	0.57	2.25	
Lualaba	1.60	0.85	2.99		1.33	0.62	2.85		1.09	0.47	2.49	
Langanyka	1.92	1.04	3.57	*	1.57	0.78	3.17		1.32	0.67	2.62	
Maniema	1.70	0.79	3.63		1.42	0.61	3.34		1.37	0.55	3.43	
Nord-kivu	2.49	1.29	4.79	**	2.04	0.97	4.29		2.09	0.98	4.47	
Bas-uele	1.30	0.68	2.50		1.48	0.65	3.37		1.07	0.47	2.47	
Haut-uele	1.41	0.60	3.32		1.56	0.62	3.91		1.40	0.55	3.52	
Ituri	1.53	0.77	3.07		1.33	0.61	2.89		1.37	0.64	2.93	
Tshopo	1.19	0.56	2.50		1.25	0.54	2.92		1.17	0.51	2.68	
Sud-kivu	3.10	1.45	6.61	**	2.57	1.15	5.73	*	2.54	1.11	5.83	*
*Intermediate factors*												
Mothers age at delivery												
< 20 years					1.00				1.00			
20–34 years					0.78	0.59	1.03		0.70	0.52	0.94	*
> =35 years					0.76	0.54	1.06		0.65	0.45	0.94	*
Mother’s body mass index												
Thin					1.00				1.00			
Normal					0.96	0.73	1.26		0.97	0.74	1.28	
Overweight					0.73	0.49	1.10	*	0.73	0.48	1.09	
Mother’s height					0.99	0.99	1.00	***	0.60	0.53	0.68	***
Birth interval												
First Birth					1.00				1.00			
< 24 months					1.17	0.86	1.59		1.38	1.06	1.79	*
> 24 months					1.05	0.79	1.40		1.23	0.95	1.59	
Family size												
Small(<4)					1.00				1.00			
Medium (4–6 members)					0.91	0.69	1.19		0.92	0.70	1.21	
Large (>8 members)					1.26	1.00	1.58	*P* = 0.05	1.18	0.93	1.48	
Number of Children												
1					1.00				1.00			
2					1.08	0.87	1.35		1.09	0.88	1.36	
3					1.21	0.93	1.59		1.21	0.92	1.58	
4 or more					0.98	0.69	1.39		0.99	0.70	1.41	
Access to safe water					0.92	0.69	1.23		0.89	0.67	1.19	
Access to hygienic toilet					0.80	0.49	1.31		0.80	0.48	1.31	
*Proximal factors*												
Birth order												
1									1.00			
2									0.74	0.49	1.13	
> =3									1.01	0.64	1.57	
Had diarrhea												
No									1.00			
Yes									1.11	0.89	1.39	
Breastfeeding within first hour												
No									1.00			
Yes									0.82	0.70	0.95	**

Model 1: Adjusted for child’s age, sex, and distal factors (mother’s education, ethnicity, place of residence and ecological zone)

Model 2: In addition to model 2, adjusted intermediate factors (mother’s age at delivery, birth interval, mother’s BMI, Mother’s height, size of family, number of children in family, access to safe water, and access to hygienic toilet)

Model 3: In addition to model 3, adjusted for proximal factors (birth order, diarrhea within past 2 weeks and initiation of breastfeeding within first hour after birth)

*OR* odds ratio, *aOR* adjusted odds ratio, *CI* confidence interval

^*^ *p* < 0.05; ^**^ *p* < 0.01; ^***^ *p* < 0.001

#### Intermediate factors

Compared to the children with mothers below 20 years of age at childbirth, children with the mothers aged 20–34 years [aOR = 0.70; 95% CI: 0.52, 0.94] and 35 years or more [aOR = 0.65; 95% CI: 0.45, 0.94] had significantly lower odds of being stunted.

In comparison to the first born children, the children who had <24 months preceding birth interval had significantly higher odds ratio of stunting [aOR = 1.38; 95% CI: 1.061.79] (Table 2, model 3).

On the other hand, mother’s Body Mass Index (BMI) and number of children within the family were not significantly associated with stunting, however model 2 detected marginally significantly high risk of stunting in children from large families [aOR = 1.26; 95% CI: 1.00, 1.58].

Environmental factors, including access to safe water and hygienic toilet had a negative association with stunting in unadjusted analysis but lost significance in the multivariate analysis.

#### Proximal factors

Results of the hierarchical modelling found age and sex consistently associated with stunting. In the final model, the adjusted odds ratio were significantly higher in every age category in comparison to <6 months children. Regarding the sex difference, the aOR among female child was significantly lower [aOR = 0.81; 95% CI: 0.70, 0.94] compared to their male counterparts.

Breastfeeding initiated within the first hour after birth was found protective against child stunting [aOR = 0.82; 95% CI: 0.70, 0.95] in the final model while birth order and history of diarrhoea showed no significant effect.

Also, the relative risk ratios (RRR) of moderate and severe stunting according to background characteristics are included as supplementary material. (See Additional file 3: Table S3).

## Discussion

In our study, we have used nationally representative data on the population of the DRC. More than 2 in every five children being stunted is a serious public health problem in the DRC. Compared to the result of Multiple Indicator Cluster Survey 2001 (MICS-2001), the prevalence has increased from 33% [34] to 43% in 2014. We found a noteworthy increment in the prevalence of stunting among the children from the poorest household from 43% in 2001 to [34] 50% in 2013/14. A large proportion of children in the DRC are also suffering from wasting (low weight-for-height) and underweight (low weight-for-age) with the figures from the DRC DHS 2013–14 being 7.9 and 22.6% respectively [5].

In our study we apply a conceptual framework for describing determinants of stunting and in our analysis we distinguish between three levels of determinants namely, distal, intermediate and proxy factors. In this discussion we assess and compare the strengths of association between stunting and different determinants. In this manner we identify key determinants and single out at what levels of determination they operate. Our findings are contrasted with the results from several other studies that have investigated determinants of stunting. This is done in order to establish an understanding of how our findings match with existing knowledge in this field.

### Distal factors

A major finding is that stunting is above all associated with factors at the distal level. First, we found a strong relationship between stunting and province and second, stunting was found to be strongly associated with socio-economic status.

In 2016 the DRC was divided into 26 provinces and we investigated stunting in these new provinces. Examining stunting in these provinces reveals that there are a number of distal factors that can be related to higher odds of stunting. Sankuru province had the highest odds of stunting. This is a landlocked province and with poor road system and no railway, the area is extremely difficult to reach. Manufactured goods including food items are sold at increasingly higher prices while cash cropping is hard to develop. Kasai is another province with a higher odds of stunting. The major livelihood in the province is artisanal mining, especially diamond mining. During the last 15 years, the diamond sector has been influenced by declining international markets. In addition, people tend to neglect food production and the province has to import much of its food. Provinces affected by war, including South Kivu, have also high odds of stunting. The land in South Kivu is fertile, but shortage of land and landlessness are problems closely related to food insecurity and chronic malnutrition. Several provinces with high odds of stunting face problems due to influx of internal as well as refugees from neighbouring countries. The DRC is characterised by widespread food insecurity and with severe food insecurity in the above provinces.

Findings from other studies have also highlighted within-country variability in terms of prevalence of malnutrition and the importance of identifying areas with particular high prevalence of malnutrition [35, 36]. The importance of analysing geographically inequalities in malnutrition is also supported by a study from the DRC using the 2013–14 DRC-DHS and data from the 11 old provinces. The study demonstrated how malnutrition in the DRC related to geographic location. A major finding was that stunting rates was highest in the provinces that rely on artisanal mining compared to the level observed in the eastern provinces affected by civil war. Our focus on the new provinces allows a focus on relatively smaller geographical units and thereby we are in a relatively good position to elicit the variety of factors that contribute to regional inequalities in stunting.

In addition to finding a strong relationship between stunting and province our research further confirms the strong relationship between stunting and socio-economic inequality. This study showed that the children living in the poorest household had higher odds of being stunted. In contrast the highest proportion of children with stunting in the DRC were categorised in the middle group in 2007 and 2010 [37, 38]. The literature has demonstrated important socio-economic inequalities in child malnutrition showing that children living in poorer households are likely to be more stunted than children in better-off households [9–11, 39]. Higher socioeconomic position is likely to represent better living conditions which again contributes to better child care and feeding practices and improved access to food and a potential decline in the occurrence of different forms of malnutrition [40].

Whereas we found strong relationships between stunting and regional and social inequalities the relationships between stunting and others factors at the distal level are unclear. This concerns the relationship between stunting and place of residence as well as the relationship between stunting and mother’s education.

We found that the largest proportion of children with stunting lived in rural households. On the other hand, in the multivariate analysis we did not find any statistically significant association between place of residence and stunting. Our findings thereby contradict findings from other studies. These studies have found that place of residence can explain variations in prevalence of child malnutrition [17, 41] and that there are evidences that in low-income countries, rural children are at a higher risk of malnutrition than their urban counterparts [42–44]. Then again our results are in accordance with research reporting that when controlling for potential confounders the odds of being stunting were not significantly different between children living in rural or urban areas [45].

We also assessed how stunting was related to mothers’ education. The crude logistic regression showed a highly significant negative correlation between mother’s education and child stunting, however, the multivariate analysis, model 2 and 3, showed that children of mothers who had secondary and higher education were less likely to be stunted than children who had mothers with no education and mothers with only primary education, but without statistical significance. Our findings apparently contrast the results of other similar studies that have demonstrated significant relationship between mother’s education and child nutrition [46–50]. On the other hand the results from a study from the DRC using DRC MICS 2001 data are uncertain [16]. It found that stunting was higher among the children whose mother had secondary education or higher. This association disappeared or appeared only after controlling for the province of residence.

### Intermediate factors

Our analysis demonstrated strong relationships between stunting and selected factors at the distal level, including regional and social inequalities. On the other hand we found the associations between stunting and variables at the intermediate level to be weaker including the association between stunting and mother’s age at delivery, birth intervals and mother’s height. With regards to access to clean water and hygienic toilet we did not find any statistically significant relationships between stunting and these factors.

Our study revealed that about one fourth of mothers were teenagers at the time of their recent delivery and the multivariate analysis showed that children who had mothers whose age was above 20 years at delivery were less likely to suffer from stunting than children with teenager mothers. Similar results were reported by a prospective study from five low-income countries [51]. Early marriage might have contributed most for a such high proportion of teenage mothers [52, 53]. Early child birth represents a health risk both for mothers and children and has negative consequences for the growth of the child [54]. During maternal growth there might be a competition of scarce nutrients between the mother and the foetus and this competition might result in early childhood undernutrition and also increase negative implications of maternal malnutrition [55].

Our research demonstrates a relationship between stunting and birth intervals and we found, in model 3, that a birth interval below 24 months was associated with statistically significant higher odds of stunting than longer birth intervals. The relationship between birth intervals and children’s nutritional status has been investigated and some studies have in accordance with our findings found short intervals to adversely affect the child’s nutritional status [56–58]. Still a review examining this relationship concluded that the reduction in stunting found in some studies could be due to residual confounding factors not covered in the analysis including breastfeeding and maternal height [59]. In our analysis we controlled for these factors and found short birth intervals to be associated with stunting.

Our findings demonstrate a higher risk of stunting among the children of mothers with short heights. Our findings thereby corroborate with previous studies showing similar results [60–62]. One explanation of maternal height to child stunting relationship is that mothers with short stature have a higher risk of giving birth of babies with low birth weight [63] which in return are at higher risk of poor growth during childhood [64].

In our study, we found children living in households with access to safe drinking water were less likely to be stunted and so were children living in households with access to hygienic toilet, but this association disappeared when we controlled for potential confounding factors. In our model 3, the strength of association was reduced and lost statistical significance. Our findings are thereby not fully in accordance with other studies in this field. These studies have found evidence that improvement in access to clean water, sanitation and hygienic facilities have a positive influence on the linear growth of children [65–67].

### Proximal factors

At the proximal level we examined several variables and their relationships with stunting including birth order, diarrhoea and early initiation of breastfeeding. Stunting was related to early initiation of breastfeeding but not the other variables. The association between stunting and early initiation of breastfeeding was in comparison with factors at the distal level weak.

Other studies have also reported early initiation of breastfeeding to protect against stunting early in childhood [68]. There are a number of benefits of early breastfeeding. Colostrum is rich in protective factors and early initiation of breastfeeding ensures that the infant receives colostrum. Studies have shown that pre-lacteal feeding increases the risk of partial breastfeeding during the first 6 months which again has been associated with stunting [69, 70].

At the proximal level, we also dealt with the issue of age of growth faltering and prevalence of stunting at different ages. Whereas the literature has focused on stunting at very early ages, our study shows that the prevalence of stunting increases further after the age of 2 years. This is in accordance with the findings of another study from the DRC analysing data from the DRC -DHS 2007 [17]. This finding highlights the important relationship between stunting and inappropriate diet also at the age above 2 years. Growth faltering at these ages could be a consequence of children above the age of 2 years being fed with food characterised by monotony and little variety.

### Nutritional policy considerations

Our findings raise a number of issues for nutritional policy consideration. The DRC nutritional policy is described in a government document from 2014 and it is this document that currently guides policy implementation [71]. The document recognises that undernutrition has many causes and that efforts to address it must be multisectoral with the government further developing partnerships with other agencies. It acknowledges the importance of decentralisation and ensuring that all parts of the country share equal access nutritional services. Particular attention is paid to vulnerable groups living in disadvantaged environments. In terms of child undernutrition, specific strategies have been developed for different age groups outlining a number of interventions required to prevent undernutrition among these groups. Examples of key areas are; promoting early initiation of breastfeeding, exclusive breastfeeding during the first 6 months, introduction of complementary feeding at 6 months, continued breastfeeding up to the age of 2 years as well as addressing micronutrient deficiencies. The results of our study support the national policy and worth mentioning is the policy’s emphasis on decentralisation, giving special attention to vulnerable groups as well as underlining the importance of early initiation of breastfeeding. There are some issues raised in our study not directly dealt with in the policy document, these include the problem of early age at delivery and short birth intervals. Furthermore we will point out the importance of policy implementation attending to the issue of within-country variability in stunting.

The government agency PRONAUT (Programme Nationale de Nutrition) is taking a lead in implementing the nutritional policy. It is present in all provinces. The organisation is especially making efforts to step up its activities in provinces that due to financial constraints receive insufficient support including seven of the new provinces.

## Conclusions

Chronic malnutrition is widespread in the DRC and comparing with previous studies there was an increase in the proportion of children suffering from stunting. We have identified key factors that can explain continued and high prevalence of child stunting. Several variables were associated with stunting and the results of our research demonstrate, in a setting with widespread child stunting, the importance of addressing determinants at the distal level. First, stunting was strongly associated with regional inequalities. These regional inequalities could be related to a number of distal factors including the province being landlocked, economic adaptation, influx of refugees and war. Second, poverty was closely connected to high prevalence of stunting with the risk of stunting being very high for children living in the poorest households. This study, therefore demonstrates the importance of giving priority to identifying pockets of stunting and geographical targeting of nutrition programmes. Geographical targeting should be combined with a strategy that addresses social inequalities in stunting and our study reconfirms the importance of nutritional interventions especially targeting vulnerable groups. In addition to identifying key factors, this study found several intermediate and proximal factors associated with stunting. These factors cover various modifiable determinants such as: early initiation of breastfeeding, short birth intervals and mother’s young age at delivery. These determinants should be incorporated in nutritional intervention programs.

## Additional files

Additional file 1: Table S1. Sample distribution and prevalence of stunting among under 5 year children according to provinces; DRC. (XLSX 13 kb)

Additional file 2: Table S2. Unadjusted odds of stunting among under five children in DR Congo 2013–14. (XLSX 14 kb)

Additional file 3: Table S3. Relative Risk Ratios for moderate and severe Stunting among under 5 year children in DR Congo 2013–14. (XLSX 17 kb)

## Abbreviations

aOR: adjusted odds ratio
BMI: body mass index
CI: confidence interval
DHS: Demographic Health Survey
DRC: Democratic Republic of Congo
HAZ: height for age z-score
MGRS: Multicentre Growth Reference Study
MICS: Multiple indicator cluster survey
OR: odds ratio
RRR: relative risk ratio
SD: standard deviation
UNICEF: United Nations Children’s Fund
WHO: World Health Organization
